# The impact of a two or three-group RCT design on blinding of patients

**DOI:** 10.1186/1745-6215-16-S2-P203

**Published:** 2015-11-16

**Authors:** Kerry Avery, Chris Metcalfe, Paul Barham, Richard Berrisford, Grant Sanders, Andrew Hollowood, Tim Wheatley, Alex Nicholson, Jenny Donovan, Jane Blazeby

**Affiliations:** 1 University of Bristol, Bristol, UK; 2 University Hospitals Bristol NHS Foundation Trust, Bristol, UK; 3 Plymouth Hospitals NHS Trust, Plymouth, UK

## 

In RCTs using subjective outcomes, blinding of patients to treatment allocation is recommended. It is unknown whether two or three-group trial designs influence blinding success. We examined the success of blinding patients within a three or two-group pilot RCT in surgery.

Cancer centres randomised patients to a three or two-group trial comparing: (i) standard open surgery; (ii) combination open and keyhole surgery, or (in one centre); (iii) totally keyhole surgery. Feasibility of blinding patients for seven days post-surgery to minimise bias in pain assessments was explored by using identical wound dressings covering all incisions. On days two and six, patients completed the Bang Blinding Index (BBI). This measures blinding success by asking patients to guess their treatment allocation. Scores range from -1 (more wrong guesses than expected) to 1 (more correct guesses), with 0 indicating perfect blinding (random guessing). Results were compared between the three and two-group studies.

The study recruited 70 patients (42 and 28 three and two-group respectively). Data indicated successful blinding in the three-group study, with fewer patients than expected guessing correctly (day two BBI scores by group (i)0.00, (ii)0.14, (iii)-0.13; day six: (i)-0.13, (ii)0.38, (iii)0.04). In the two-group study, slightly more patients became unblinded, with more than expected guessing they had combination surgery (day two: (i) -0.73, (ii) 0.35; day six: (i) - 0.25, (ii) 0.57).

This pilot study successfully blinded patients in a three-group study. However, in the two-group study more patients had become unblinded. This suggests that blinding is more successful in multi-arm studies.

